# Endoscopy-assisted resection of a sphenoid-wing meningioma using a 3D-printed patient-specific pointer in a dog: A case report

**DOI:** 10.3389/fvets.2022.979290

**Published:** 2022-11-16

**Authors:** JaeWon So, HaeBeom Lee, JaeMin Jeong, Franck Forterre, YoonHo Roh

**Affiliations:** ^1^Department of Clinical Sciences, College of Veterinary Medicine, Chungnam National University, Daejeon, South Korea; ^2^Division of Small Animal Surgery, Department of Clinical Veterinary Medicine, Vetsuisse-Faculty, University of Bern, Bern, Switzerland

**Keywords:** meningioma, sphenoid wing, skull-base tumor, endoscope, 3D PSP, CSF leakage

## Abstract

A 9-year-old female mixed-breed dog presented for treatment of a presumed sphenoid-wing meningioma. Clinical signs included tonic-clonic seizures lasting <1 min, which had started 3 months previously. The physical examination results were unremarkable. An eccentrically located neoplastic cystic structure in the right sphenoid bone region suggestive of a meningioma and peritumoural brain oedema was observed in pre-operative magnetic resonance imaging (MRI). Prior to surgery, a three-dimensional (3D) patient-specific pointer (PSP) was designed using computed tomography (CT) images and computer-aided 3D design software. After a targeted approach and exposure of the lateral part of the right temporal lobe by a craniectomy guided by the 3D-PSP, complete macroscopic piecemeal resection of the meningioma could be performed using endoscopy-assisted brain surgery. Post-operative MRI confirmed complete excision of the tumor. Anticonvulsive therapy was discontinued after 90 days, and the dosage of anticonvulsants was tapered 2 weeks after surgery. At a follow-up examination 225 days post-operatively, recurrence of seizures was not observed, and the absence of tumor recurrence was confirmed by a repeat MRI examination. To the best of our knowledge, this is the first report in veterinary medicine describing a successful resection of a sphenoid-wing meningioma using a 3D-PSP. 3D-PSP-assisted craniectomy may be a surgical option for some canine skull-based tumors, such as sphenoid wing meningiomas.

## Introduction

Meningiomas are the commonest primary intracranial canine tumors, especially in large-breed dogs, representing 49–51.5% of all intracranial neoplasms (1–3). Meningiomas can occur at many sites, including the olfactory/frontal region, the skull-base cavity, and the suprasellar and parasellar regions. However, skull-base meningiomas are rare in dogs and cats (2, 4), and the prognosis of these tumors depends on several factors such as location and therapeutic strategy (2–6). Most meningiomas in humans are treated with complete surgical resection, and the recurring tumors are treated with chemotherapy and/or radiotherapy (2, 3, 7). However, pre-operative brain biopsies and complete perioperative surgical removal of tumors are challenging, and few reports describe their use in veterinary medicine (6, 8).

Pre-operative surgical planning and accurate perioperative surgical techniques are essential to achieve good functional results (7, 9, 10). However, surgery of the canine skull base is challenging because of the complexity of the vessels and nerves in this region (10–12). Determination of the safest and most accurate approach is essential to minimize permanent surgical trauma to the normal brain (9, 12, 13). Neuronavigation (NN) and the endoscopic approach have been developed to increase the accuracy and safety of surgeries (10, 14, 15). However, NN has several limitations when applied to canines. NN can interfere with the surgical approach due to the relatively small size of the canine skull. Cost also plays a key role in limiting its adoption in veterinary medicine (9, 10, 15–17). Recently, three-dimensional (3D) printing has been widely used to overcome these limitations in neurosurgery (9, 12, 18). 3D-printed patient-specific skull-contoured brain biopsy guides have reportedly aided needle placement into the target in dogs (8). 3D patient-specific drill guides have also been reported to be widely used for accurate spinal screw placement (18, 19).

Very few reports of veterinary skull-base meningiomas seem to exist (8, 10, 15). Moreover, unlike human medicine, no specific surgical strategies are available for individual tumor sites in dogs. Here, we describe (1) the feasibility of targeting sphenoid-wing meningiomas using a fabricated 3D-printed patient-specific pointer (3D-PSP), and (2) the endoscopy-assisted surgical procedure that overcomes the limitation of a complex and challenging surgical site.

## Case description

### Case

A 9-year-old, female mixed-breed dog with a body weight of 15.1 kg and a body condition score of 5/9 was referred for generalized seizures and meningioma. The reported clinical signs included tonic-clonic seizures lasting <1 min, which had started 3 months prior to presentation, and decreased appetite. Hematological and blood investigation results were within normal limits except for the liver panel findings. The levels of alanine transaminase, alkaline phosphatase, and γ-glutamyl transferase levels were mildly elevated.

**Figure 1:**
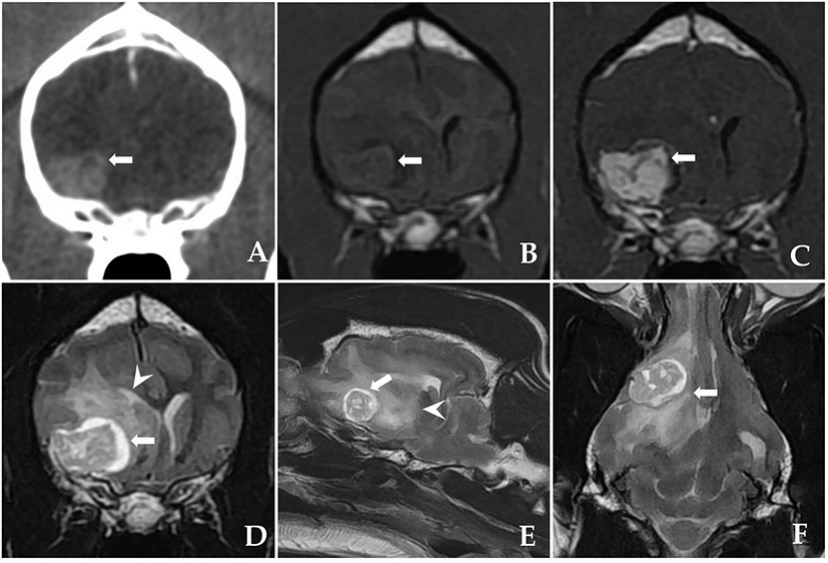
Postcontrast computed tomography (CT) and pre-operative magnetic resonance imaging (MRI) showing a sphenoid-wing meningioma with a large eccentrically located cystic structure in a dog. **(A)** A postcontrast transverse CT image: the mass is intensely and uniformly enhanced. The mass is well-marginated and broad-based, indicating an extra-axial origin. **(B)** T1-weighted (T1W) transverse image: an isointense mass is discovered under the right sphenoid wing. **(C)** Post-contrast T1W transverse image and T2-weighted (T2W) images showing **(D)** transverse, **(E)** parasagittal, and **(F)** dorsal heterogenous contrast-enhancement (arrow) with intratumoural accumulation of fluid and peritumoural oedema (arrowhead).

At presentation, the physical and neurological examinations were normal. The computed tomography (CT) and magnetic resonance imaging (MRI) examinations performed at the referring veterinary clinic had discovered a large eccentrically located cystic structure under the right sphenoid wing. The marked uniform contrast enhancement of the heterogeneous cystic remainder was confirmed on CT and MRI. In addition, contrast enhancement revealed well-defined, broad-based tumor margins conforming to the meningeal plane. This structure showed heterogeneous, intratumoural fluid accumulation characterized by an enlarged, sharply defined outer margin with a significant hyperintense signal and peritumoural oedema on T2-weighted images. Based on the imaging characteristics (Figure 1), cystic meningioma was suspected. Neither thoracic radiography nor abdominal ultrasonography revealed any abnormalities.

### Choice of surgical technique

**Figure 2:**
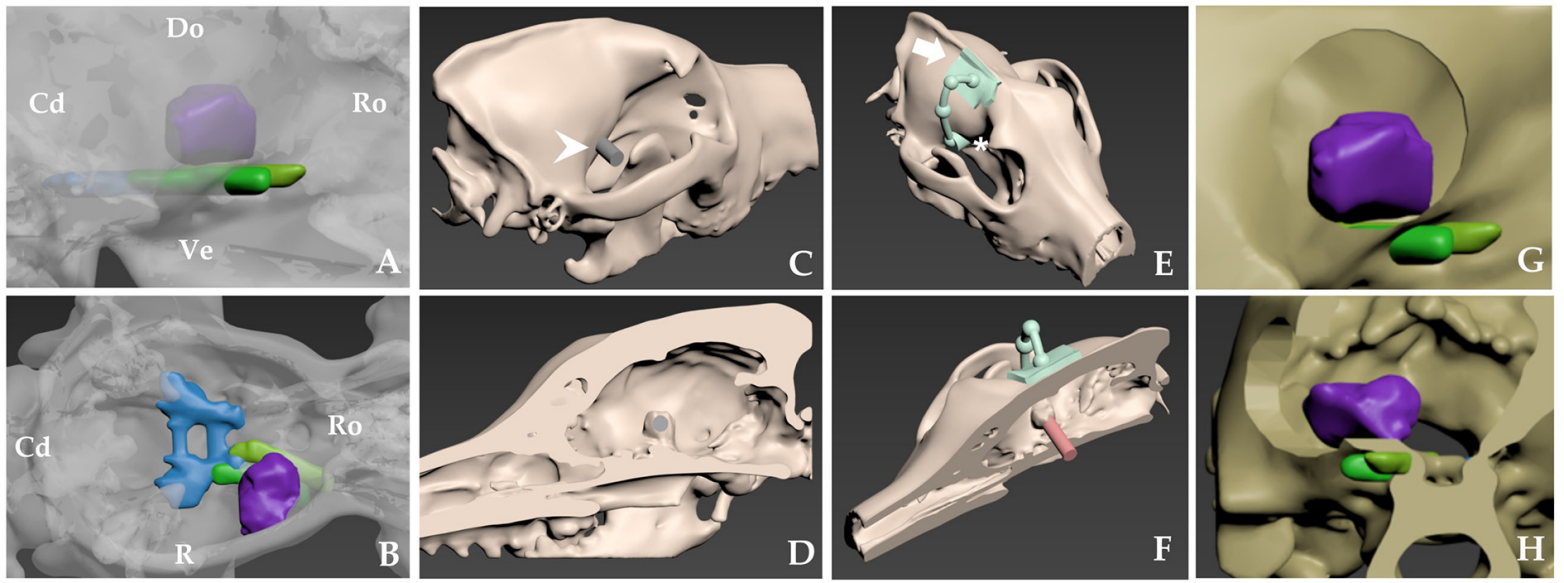
Schematic images of the approximate location of tumor-related neurovascular structures and pre-operative skull-contoured three-dimensional (3D) patient-specific pointer (PSP) design process for craniectomy of a right sphenoid-wing meningioma. **(A,B)** Skull rendering is based on computed tomography (CT) scans. Tumor, purple; middle cavernous sinus, blue; optic nerves, yellow; orbital-fissure nerves, including the oculomotor nerve, the trochlear nerve, the ophthalmic nerve, and the abducens nerve, green. **(C,D)** The tumor is confirmed in a 3D bone model with a sagittal cut of the head. A 3D tumor-targeting gray cylinder (white arrowhead) the same size as the tumor passes through the sphenoid-wing bone representation. **(E)** The attachment arm (white arrow) and pointer (asterisk*) are shown. The diameter of the pointer is twice that of the gray cylinder **(C,D)**. **(F)** Attachment of the bone model and the PSP at the sagittal plane indicates the tumor's location. The red cylinder shows the relationship between the PSP and the tumor. **(G,H)** After the craniectomy, the expected relationship of surgical window and nerves in lateral **(G)** and transverse images **(H)**. Cd, caudal; Do, dorsal; Lt, left; Ro, rostral Rt, right; Ve, ventral.

The lesion resulted in mass effect, intracerebral oedema, and associated signs such as generalized seizures, which were indications for intervention in this case. CT was used to measure the extent of bony invasion or hyperostosis due to the possibility of the tumor infiltrating the skull bone. T2-weighted MRI imaging identified intraparenchymal oedema and important structures such as the cavernous sinus and optic nerve. Schematic images were prepared for pre-operative planning using CT and MRI, which demonstrated the approximate relationships among the tumor, cerebral arteries, optic nerve, and bony landmarks (Figures 2A,B). The tumor was identified as sphenoid-wing meningioma of the skull base. A craniectomy with dura mater resection was planned.

In human medicine, lateral and middle sphenoid-wing meningiomas can be resected using pterional craniotomy, but this is a challenging technique due to closely located neurovascular structures such as the optic nerve, the cavernous sinus, and the middle cerebral artery (7). The relationships between the vascular and bone morphology and the anatomic landmarks have been clarified in human medicine, but little information of this kind is available to assist sphenoid-wing meningioma surgery in veterinary medicine (7). Dogs have relatively massive temporal muscles and different sizes, locations, and morphologies of neurovascular structures and structures such as the zygomatic arch and the coronoid process of the mandibular bone (20–23). Therefore, for accurate and safe delineation of the surgical site, we decided to employ a 3D-printing technique similar to the use of a 3D-printed biopsy guide, since we lacked NN capability and sufficient experience with traditional *in-silico* planning and *in-vivo* measuring techniques.

### Planning and production of the 3D-PSP

Surgical planning, including confirmation of the location, size, and shape of the 3D-PSP and identification of the exact location of the tumor, was performed using computer-aided 3D design software (3-DS Max; Autodesk, San Francisco, CA, USA) applied to the MRI and CT images (20, 21). This was necessary because a PSP used in brain surgery can differ in detail from patient-specific guides used on the spine. Detailed guide concepts and methods have been described previously (20, 21).

Postcontrast CT images (Alexion™; Canon Medical Systems Corporation, Otawara, Japan; slice thickness, 1 mm; operating parameters, 120 kV and 12 mA) of the patient were obtained in the DICOM file format (Digital Imaging and Communication in Medicine) and converted to stereolithography files. Segmentation, 3D model reconstruction, and prosthesis design were then performed using computer-aided design software (Mimics; Materialize NV, Leuven, Belgium). The design process required ~1 h (20).

The tumor was confirmed in a sagittal cut of the 3D bone model using the computer software. A 3D tumor-targeting cylinder passing through the sphenoid wing bone was created, and the diameter and location of the cylinder were designed to match those of the tumor (Figures 2C–H). The diameter of the resulting pointer was twice that of the cylinder. The footprint of the pointer represented the margins of the planned craniectomy, and allowed sufficient surgical space for tumor resection. An attachment arm was created for the PSP, which was designed to fit the most characteristic surface of the frontal bone and sagittal crest. The surface of the arm had an inverted skull structure to ensure stable bone attachment. Once the arm was fixed, the pointer automatically indicated the craniectomy site under which the tumor was located.

To ensure accurate surgical planning, rehearsal surgery was performed using a 3D-printed bone model and the PSP. Both were printed using the RS6000 3D printer (Uniotech, Shanghai, China) according to a previously described protocol (20). Printing and sterilization of the 3D bone models and the PSP prior to the real surgery required ~1 day (20).

### Surgical technique

Midazolam (0.1 mg/kg IV; Midazolam®, Bukwang Pharmaceutical, Seoul, Korea) and propofol (6 mg/kg IV; Propofol®, Myungmoon Pharm, Seoul, Korea) were used to induce anesthesia. After intubation, oxygen and total intravenous anesthesia were used to maintain anesthesia prior to durotomy. The drugs used for total intravenous anesthesia were propofol [0.05–0.5 mg/kg/min, constant-rate infusion (CRI); Propofol®, Myungmoon Pharm] and remifentanil (0.1–0.3 μg/kg/min CRI, Remiva®, Hana Pharm, Seoul, Korea). Anesthesia was maintained with isoflurane (0.7–1.0%; Ifran®, Hana Pharm, Seoul, Korea) post-durotomy. Plasmalyte (2 mL/kg/h IV; Plasma Solution-A®, HK inno.N, Cheongju, Korea) was administered throughout the surgery. Prior to surgery, the dog received phenobarbital (3 mg/kg PO; Phenobarbital®, Hana Pharm, Seoul, Korea), prednisolone (0.5 mg/kg PO; Solondo®, Yuhan Corporation, Seoul, Korea), dexamethasone (0.2 mg/kg IV; Dexamethasone® Jeil pharmaceutical, Seoul, Korea), cefazoline (22 mg/kg IV; Cefazolin In®, Chong kun dang healthcare, Seoul, Korea), maropitant (1 mg/kg SC; Cerenia®, Zoetis, New jersey, US), and mannitol (0.5 g/kg IV; D-mannitol inj®, Dai Han Pharm, Seoul, Korea).

**Figure 3:**
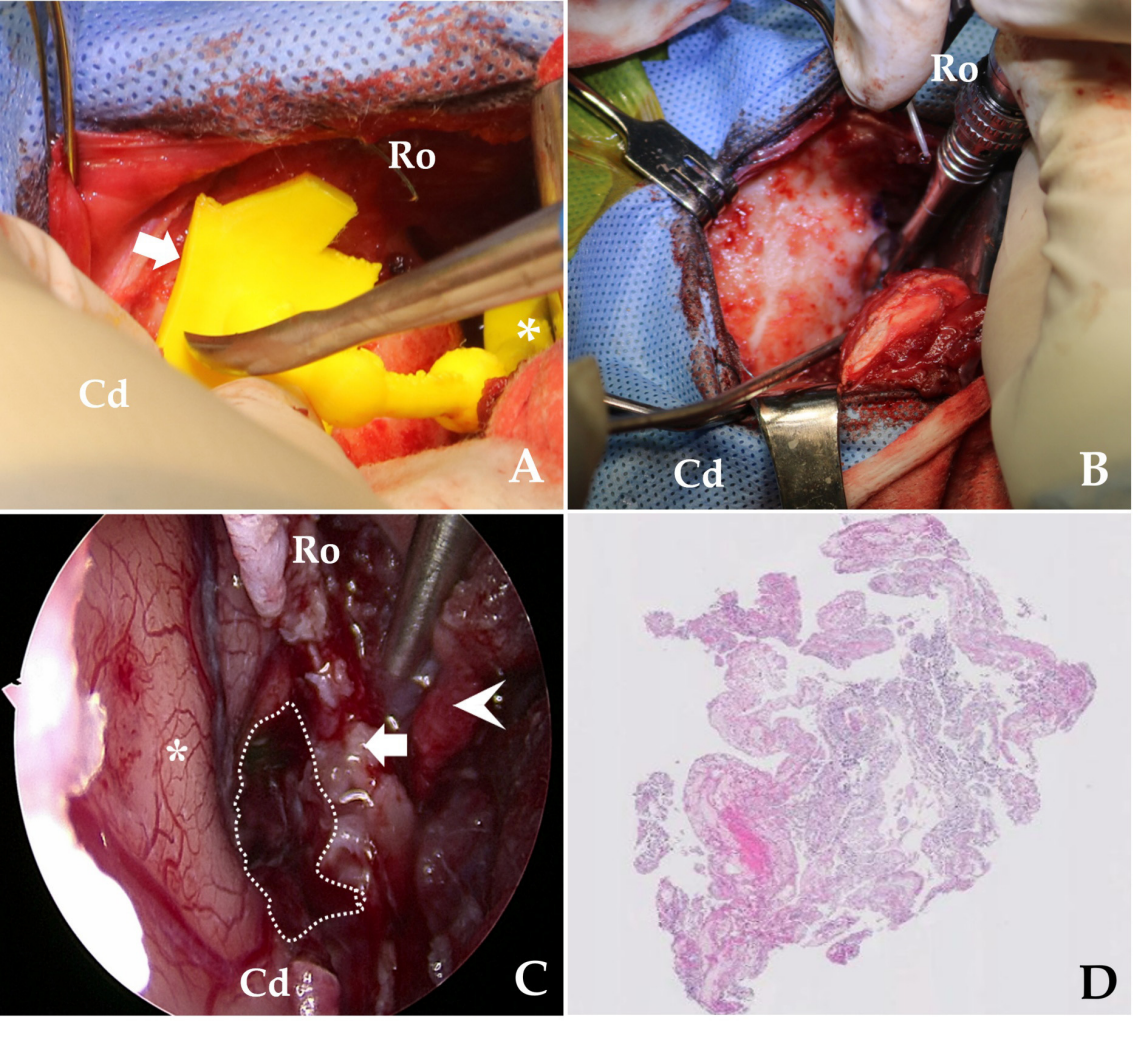
Overview of the skull-contoured 3D-printed PSP applied to the patient. **(A)** The pre-operative, skull-contoured attachment arm of the 3D-PSP (white arrow) is mated to the bone, and the pointer (asterisk, *) then indicates the craniectomy site above the tumor. **(B)** After outlining the drilling site using the pointer, the craniectomy is performed. **(C)** Intraoperative endoscopy image after removal of the meningioma showing the brain (asterisk, *), bone (white arrow), muscle (arrowhead), and tumor resection site (dotted line). Complete, macroscopic piecemeal resection of the tumor is confirmed by endoscopic imaging. **(D)** Photomicrograph demonstrating the histopathological criteria for diagnosing a meningioma. On microscopic examination, neoplastic cells are characterized by indistinct cell borders, moderate amounts of eosinophilic cytoplasm, and an oval, centrally placed nucleus with clumped to finely stippled chromatin and a single nucleolus (H&E, X400). Cd, caudal; Ro, rostral.

The surgical procedure was based on sphenoid-wing craniectomy (22–24). The fur was clipped from the lateral canthus of the eyes to the occipital protuberance, and laterally to the zygomatic arches. The skin was aseptically prepared with chlorhexidine and disinfected with alcohol and povidone-iodine. The dog was placed in sternal recumbency with its head elevated to ~30°. A 6-cm vertical scalp incision was made dorsal to the zygomatic bone. The platysma was incised vertically. The superficial temporal nerve and rostral auricular nerve were retracted caudally, and the zygomatic branch of the facial nerve was retracted cranially. The zygomatic attachment of the temporal muscle was incised and bluntly elevated from the temporal bone. The scalp and temporal muscles were turned over and fixed laterally using a Senn and Gelpi retractor, and a wide spatula was used to maintain the surgical field (22–24). After separating the muscle per requirement, the pre-made 3D-PSP was applied to the skull landmarks described in Section Planning and Production of the 3D-PSP without complete removal of the periosteum (20) (Figure 3A). After dissection of the soft tissue of the skull, the craniectomy site was determined based on the 3D-PSP (Figure 3B) (20). The craniectomy line was made along the outer boundary of the pointer using a surgical pen and electrocautery. The bone was resected using a 2.0 mm bone burr (2.0 mm burr/drill; Stryker Corp., Kalamazoo, MI, USA) along a tool path indicated by the 3D PSP, following which the tumor could be immediately identified (20, 23).

**Figure 4:**
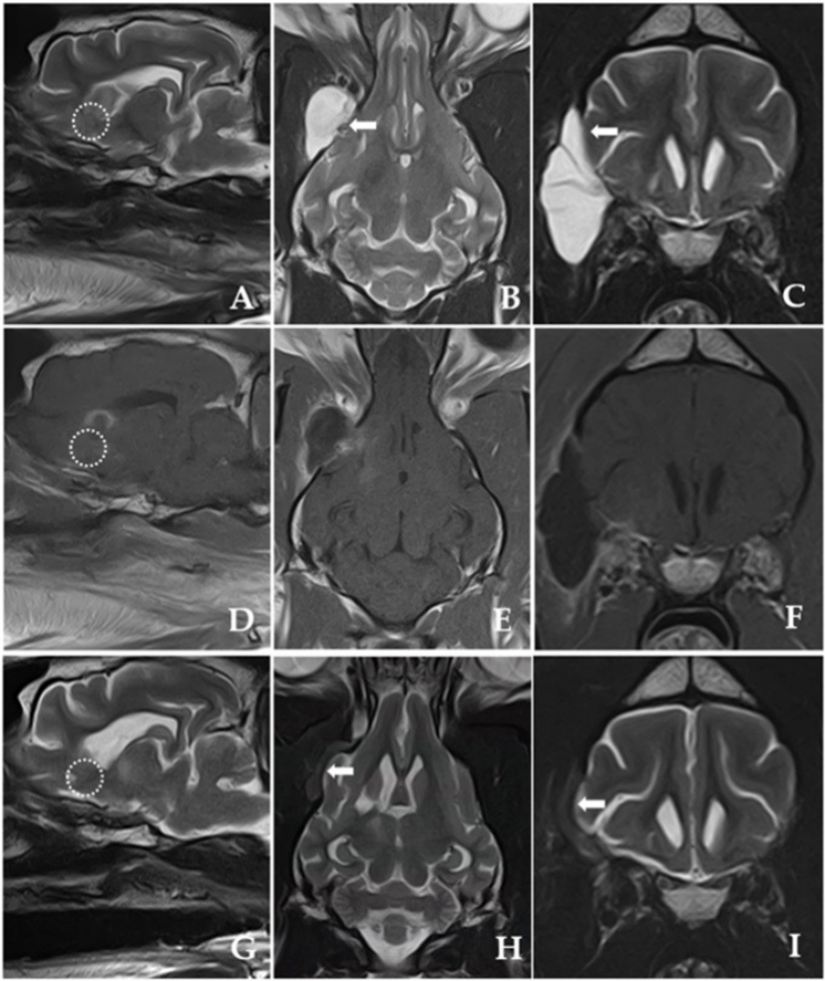
Post-operative T2-weighted and post-contrast T1-weighted MRI images obtained 18 days after the surgery **(A–F)**. **(G–I)** Shown are post-operative T2-weighted MRI images 225 days after the surgery. **(A,D,G)** Shown are parasagittal images of complete resection of the tumor (dotted line) with well-managed peritumoural brain oedema after surgery. [**(B,C)**; white arrow] Marginal brain tissue herniation accompanying a fluid-filled lesion is discovered outside the right frontal lobe with a hyperintense signal presumably due to cerebrospinal fluid (CSF) leakage immediately after the surgery. T2-weighted MRI images obtained 225 days post-operatively [**(H,I)**; white arrow] show evidence of CSF leakage improvement, but the volume of brain tissue herniating through the dural defect remains and slightly increases.

After exposing the tumor using the pointer, we maximized visualization of the surgical site using an endoscope. Under endoscopic visualization, durotomy was performed using a #11 scalpel and micro-scissors, and the ventrolateral aspect of the lateral temporal lobe was exposed under an enhanced surgical field (20, 22, 23). To magnify the surgical site, a 10-mm, 0° rigid telescope was connected to a high-definition camera system (Stryker Endoscopy, Stryker Corp., Kalamazoo, MI, USA). A mechanical endoscope holder was used to maintain the position of the endoscope. The tumor margins were then carefully dissected away from the normal brain tissue until macroscopic, piecemeal resection was complete (Figure 3C). Although the location of neurovascular structures was not easily identified, there was no significant bleeding. Following tumor removal, we applied Floseal® (Baxter Healthcare Corporation, Fremont, CA, USA) (25). After confirming complete haemostasis of minor bleeding, the craniectomy area was covered with temporal muscle. The incised temporal and masseter muscle, subcutaneous tissues, and skin were closed per set guidelines (22, 25). A biopsy specimen was removed from the tumor and fixed in 10% neutral-buffered formalin for histopathological examination. Eighteen-day post-operative MRI confirmed that the tumor was totally removed (Figure 4).

### Outcomes, complication, and follow-up

Post-operatively, temperature, pulse, respiratory rate, capillary refill time, blood pressure, auscultation, and neurological status were monitored. Post-operative analgesia was provided with remifentanil (5 μg/kg/h CRI; Remiva®, Hana Pharm) while observing the patient's pain reaction for the first 12 h. The analgesia was terminated the next day, and the patient was encouraged to walk. Hartmann's solution (2 mL/kg/h CRI) was administered for 24–36 h. The patient received cefazoline (22 mg/kg, IV every 12 h; Cefazolin ®, Chong Kun Dang Healthcare) for the first post-operative week, and phenobarbital (2 mg/kg, PO every 12 h; Phenobarbital®, Hana Pharm) was tapered over 3 months post-operatively. The clinical features and the results of histopathological examination of the surgically resected tissue samples were suggestive of a grade 1 meningothelial meningioma (Figure 3D).

Thirty-two days after surgery, the patient was readmitted to our clinic because of fluid fluctuations at the surgical site. The owner reported that the patient repetitively scratched the surgical site at home. Ultrasound was used to discriminate the fluid, and the presumptive diagnosis was seroma. The fluid was red, and analysis showed a haematocrit level of 4.5% and total nucleated cell count of 24,910 cells/μL. The seroma responded well to surgical debridement and application of a Barovac drainage tube (Barovac® Sewoon Medical, Seoul, Republic of Korea), which provided continuous negative pressure.

The follow-up observations comprised repeated neurological and MRI examinations for 296 days post-surgery. Recurrence of seizures was not observed, and complete tumor resection with resolution of peritumoural brain oedema was confirmed by repeated MRI (Figure 4).

## Discussion

This is the first case report describing a feasible, endoscopy and 3D-PSP–assisted, sphenoid-wing meningioma resection in a canine patient. The PSP allowed us to perform the craniectomy targeting the brain tumor. Complete macroscopic piecemeal resection of the sphenoid-wing meningioma was performed accurately without injuring the main blood vessels. The patient had a good clinical outcome without significant complications over 296 days of follow-up.

Sphenoid-wing meningiomas are a type of skull-base tumor (2, 4). There are extremely limited reports of these tumors in dogs and cats. In humans, complete surgical resection is the recommended treatment (2, 3, 7). However, surgical treatment is an arduous task for several reasons. First, skull-base tumors are located deep under the brain; thus, it is challenging to target the tumor accurately and perform surgical resection in a limited operative space (10, 11, 26, 27). In addition, several arteries and veins are present in complex areas of the skull base, and the restricted field of approach makes it difficult for the surgeon to perform complete tumor resection without vessel injury (22). Neurosurgical planning is vital to determine a precise and safe surgical approach for minimizing brain damage in patients with skull-base tumors because of the closely located, critical neurovascular structures (9, 12, 13). Therefore, the surgeon must be familiar with vascular and bone morphology as well as with anatomy. Visual interpretation of conventional MRI scans is usually sufficient for diagnosis, but the planning and execution of neurosurgical procedures require transformation of these data into a 3D space (1–4, 9, 28). In humans, NN has improved the 3D localization of brain tumors (10, 14). NN captures vital neurovascular structures as well as critical neighboring structural and functional regions, and converts this information into digitized neuroradiological data. An optimized approach is determined through this simulated presurgical step, which can significantly facilitate skull-base surgeries (14). Furthermore, recent frameless NN systems have improved the usefulness of NN in skull-base tumor surgeries (17). In veterinary medicine, NN has reportedly allowed surgeons to approach the pituitary fossa with clinically acceptable accuracy (29). NN has also been used to obtain diagnostic brain biopsy samples (8, 30). However, the relatively small size and thickness of the canine skull compared to humans makes it difficult for neurosurgeons to apply NN accurately in dogs (22, 23, 31); attempting NN in the presence of a large volume of temporal muscle and zygomatic bone could result in longer access times and narrower surgical fields. These limitations make it difficult for veterinary neurosurgeons to apply NN to sphenoid-wing meningioma resection. However, 3D-printed biopsy guides have been recently used for veterinary medicine without NN and have proven feasible in terms of outcomes (8).

3D-printing technology offers significant advantages for neurosurgery. 3D-printed material can facilitate presurgical planning by converting visual images into tactile models (18). Surgeons can rehearse realistic surgery because 3D-printed materials can be milled, cut, and drilled with the usual surgical instruments as often as required (18). Customized prosthetics, implants, fixtures, and 3D-printed biocompatible materials have been widely used recently (9, 12, 18). This 3D-printing technique can have several advantages when applied to brain surgeries (8, 12, 20). 3D printed guide techniques such as 3D brain biopsy can designate the surgical location more accurate and less-invasive (8). When resecting brain tumors, precise surgical site exposure is crucial to avoid excessive manipulation of the brain parenchyma (6, 8–13, 32, 33). If the surgical window is inaccurately delineated at the first time, expansion of the surgical window size would be inevitable (20). Moreover, it can be applied to the hands of less experienced surgeons (8, 20, 34). Surgeons with 3D PSP can be pre-operatively accustomed to patient specific circumstances, such as neurovascular structures and anatomic bony landmark in the course of 3D planning (12, 34). Also surgeons would reduce the surgical time because they can skip the setting of the neuronavigation system and special imaging instruments perioperatively (8, 20, 30). In the present case, the exact craniectomy site could be easily and safely determined, avoiding in-surgery modification of the surgical window. The 3D-printed implant fitted perfectly to the skull surface only at the predefined craniectomy site; any slight displacement of the print would have led to an incongruency between the under-surface of the print and the surface of the skull, thereby indicating inaccurate positioning. The positioning of the 3D print was also limited by the narrow and deep approach to the sphenoid bone (20–22, 25, 26). However, in close-up positions, an endoscopy-assisted approach can provide a clear view of the surgical view (23). This advantage is beneficial during surgical procedures for deep-seated lesions in narrow spaces (15, 22, 35). Therefore, a 3D-PSP combined with an endoscope can facilitate the complex procedure of sphenoid-wing meningioma resection in small-breed dogs.

In human medicine, the surgical management of sphenoid-wing meningioma is reportedly challenging because of the deep and narrow surgical site in the skull base and the proximity of critical neurovascular structures such as optic nerves and arteries (7, 23, 29). In this case, the surgical approach, surgical view, and space for instruments were insufficient due to the tension around the temporal muscle and the coronoid process of the mandibular bone. Gross total resection requires elevation of all the tumor-infiltrated tissues, including the dura mater, to avoid tumor recurrence (36). Here, after tumor resection, the craniectomy window was covered with the temporal muscle; the dura mater could not be repaired with artificial graft because the working space was insufficient (35, 36). MRI showed cerebrospinal fluid (CSF) leakage with evidence of a CSF fistula outside the right frontal lobe 18 days post-operatively, which significantly improved 225 days post-operatively (Figure 4). In humans, CSF leakage is reportedly a complication of cranial and spinal surgery, especially when the final structural repair of the dural defect is incomplete (37–39). Accordingly, it is reasonable to presume that the CSF leakage in our patient was caused by the dural defect. CSF leakage has been associated with secondary complications such as meningitis, encephalitis, and wound infection, and thus various treatment options have been established for persistent CSF leakage in humans, including conservative therapy, epidural blood patches, and surgical patches (39–41). However, reports describing treatments for CSF leakage in animals are rare (40, 41). Our patient was treated conservatively, as several studies in humans have suggested conservative treatment for almost all CSF leaks (42, 43). In our case, it remained unclear if the seroma observed after 32 days was associated with the initial CSF leak.

This study had some limitations. First, the duration of time required to design and print the 3D-PSP models was relatively long (8, 18, 19). This represents a significant limitation on the use of this model in emergent situations such as intracerebral hemorrhages. Second, the surgeon's technique has a major influence on accurate pointer positioning. To ensure a perfect fit, the surgeon must separate the soft tissue sufficiently for adequate exposure of the area where the pointer will be applied (19). Slight deviations of the pointer during surgery can have serious consequences such as introducing errors in the craniectomy site (20). Improvements in software and adequate surgeon experience would increase the effectiveness of 3D-PSP applications. Third, we have included the findings of only one dog, although the feasibility of 3D-PSP-aided complete resection of sphenoid-wing meningioma has been demonstrated. Fourth, we could not evaluate the location or size of the surgical window, as previous studies have done, because post-operative CT was not performed (20, 21). Although the 3D-printed pointer can be modified to fit the patient, further research is necessary to compare the accuracy and utility of this method with current best practices. Further studies with larger samples of dogs are also warranted to simplify the pointer manufacturing process and ensure the accuracy, reproducibility, and reliability of this technique. The development of additional methods and techniques is required to avoid damage to critical vessels such as the middle cerebral artery during resection of skull-base tumors. In humans, brain angiography has been successfully applied prior to surgery to determine the meningioma's relationship to the surrounding vasculature and their degree of encasement. However, reports describing these tumors in veterinary medicine have been very few and application of angiography proved impossible in the present case due to the owner's financial constraints (36, 44). Further studies on the use of MRI- and CT-based brain angiography in veterinary medicine are warranted.

## Conclusions

We demonstrated that sphenoid-wing meningioma could be safely approached by endoscopy-assisted 3D-PSP without requiring NN. Clear visualization resulted in complete resection of the skull-base meningioma. The described method is a viable option for performing skull-base craniectomy in dogs.

## Data availability statement

The original contributions presented in the study are included in the article/supplementary material, further inquiries can be directed to the corresponding author/s.

## Ethics statement

Ethical review and approval was not required for the animal study because was a case report. We did the surgery. Written informed consent was obtained from the owners for the participation of their animals in this study. Written informed consent has been obtained from the owner of the animal to publish this paper.

## Author contributions

JS, YR, and HL managed the case and wrote and edited the manuscript. HL, YR, and JS performed the surgeries. YR and FF critically reviewed and revised the manuscript. JJ supervised the clinical management of the patient. All authors contributed to the preparation of the manuscript and have approved its publication.

## Conflict of interest

The authors declare that the research was conducted in the absence of any commercial or financial relationships that could be construed as a potential conflict of interest.

## Publisher's note

All claims expressed in this article are solely those of the authors and do not necessarily represent those of their affiliated organizations, or those of the publisher, the editors and the reviewers. Any product that may be evaluated in this article, or claim that may be made by its manufacturer, is not guaranteed or endorsed by the publisher.
